# ROAR-CAT: Rapid Online Assessment of Reading ability with Computerized Adaptive Testing

**DOI:** 10.3758/s13428-024-02578-y

**Published:** 2025-01-14

**Authors:** Wanjing Anya Ma, Adam Richie-Halford, Amy K. Burkhardt, Klint Kanopka, Clementine Chou, Benjamin W. Domingue, Jason D. Yeatman

**Affiliations:** 1 Stanford University Graduate School of Education, 520 Galvez Mall, Stanford, CA 94305 USA; 2 Division of Developmental Behavioral Pediatrics, Stanford University School of Medicine, Stanford, CA USA; 3 Department of Psychology, Stanford University, Stanford, CA USA; 4 Present Address: New York University, New York, NY USA; 5 Cambium Assessment, Washington, DC USA

**Keywords:** Dyslexia, Screening tools, Psychometrics, Computerized adaptive testing, Lexical decision task, Word recognition

## Abstract

The Rapid Online Assessment of Reading (ROAR) is a web-based lexical decision task that measures single-word reading abilities in children and adults without a proctor. Here we study whether item response theory (IRT) and computerized adaptive testing (CAT) can be used to create a more efficient online measure of word recognition. To construct an item bank, we first analyzed data taken from four groups of students (*N* = 1960) who differed in age, socioeconomic status, and language-based learning disabilities. The majority of item parameters were highly consistent across groups (*r* = .78–.94), and six items that functioned differently across groups were removed. Next, we implemented a JavaScript CAT algorithm and conducted a validation experiment with 485 students in grades 1–8 who were randomly assigned to complete trials of all items in the item bank in either (a) a random order or (b) a CAT order. We found that, to achieve reliability of 0.9, CAT improved test efficiency by 40%: 75 CAT items produced the same standard error of measurement as 125 items in a random order. Subsequent validation in 32 public school classrooms showed that an approximately 3-min ROAR-CAT can achieve high correlations (*r* = .89 for first grade, *r* = .73 for second grade) with alternative 5–15-min individually proctored oral reading assessments. Our findings suggest that ROAR-CAT is a promising tool for efficiently and accurately measuring single-word reading ability. Furthermore, our development process serves as a model for creating adaptive online assessments that bridge research and practice.

**Supplementary Information:**

The online version contains supplementary material available at 10.3758/s13428-024-02578-y.

## Introduction

Accurately assessing students' reading ability and efficiently identifying struggling readers is a shared challenge faced by researchers, clinicians, teachers, and school administrators. The Rapid Online Assessment of Reading (ROAR) is a web-based lexical decision task that has shown promise in measuring reading abilities without the need for a proctor, with comparable results to traditional, individually administered reading assessments (Yeatman et al., 2021). The initial version of ROAR used a full item bank with items presented in random order, and the assessment took about 20 min to complete. Previous research applied item response theory (IRT; Lord, 2012) and found that the one-parameter logistic model (1PL), with a fixed lower asymptote of 0.5 (see Eq. 1), was a good fit. This model estimates the probability of a correct response based on the item’s difficulty (*b*) and the individual’s latent reading ability (theta, *θ*). Here, we ask whether computer adaptive testing (CAT) techniques can be used to create a more efficient online lexical decision task. ROAR-CAT is designed for two purposes: (a) to be an efficient, reliable, and generalizable research tool, and (b) to serve as an effective screening tool for reading difficulties, including dyslexia, that can be implemented at-scale in schools.

1 $$P\left({X}_{i}=1\left|\theta \right.\right)=0.5+0.5\frac{exp\left(\theta -{b}_{i}\right)}{1+exp\left(\theta -{b}_{i}\right)}$$

Several questions must be answered before we can ensure that ROAR-CAT fulfills these use cases. First, are the difficulty estimates of individual ROAR items invariant across different school populations? Even though lexical decision tasks are widely used in cognitive science research, item responses have not been directly compared across diverse populations of school-age children to ensure that item difficulty is consistent in children with different educational experience and background knowledge. Second, can we make a more efficient version of ROAR that combines IRT and CAT to efficiently and reliably measure reading ability across a broad age range spanning kindergarten through high school? The initial version of ROAR presented items in a random order, and the efficiency and potential bias of a computer adaptive lexical decision task has not been studied. Third, how closely is ROAR-CAT related to other proprietary reading/dyslexia screening tools commonly used in schools? While there is a wealth of measures commonly used in practice, many of these have not been systematically studied in research. In this paper, we present a series of three studies designed to address these research questions. It is also important to note that while ROAR is an English-language assessment developed in the United States, the following questions and proposed methods can be adapted for use in other languages and regions.

### Lexical decision task as a measure of reading ability

The lexical decision task (LDT) requires participants to decide whether combinations of letters are words or not. It is a significant tool in cognitive science for exploring cognitive processes related to visual word recognition. In recent times, researchers have begun to investigate the relationship between LDT performance and reading ability indexed by standardized measures that involve reading out loud. Studies by Martens and de Jong (2006) and Araújo et al. (2014) have shown that LDT response times (RTs) differ between children with dyslexia and those with typical reading skills. Specifically, these studies identified larger word length and lexicality effects in children with dyslexia than in those with normal reading abilities. In a separate study, Sabatini and colleagues (2019) designed a multiple-choice test in which participants are asked to decide whether what they see (a) is a real word, (b) is not a real word, or (c) sounds exactly like a real word, and ran a validation study in schools. In a follow-up study, Wang and colleagues (2020) observed that though poor decoders generally exhibit lower decoding efficiency, reflected by extended processing times in recognizing real words and pseudo-homophones, they take significantly less time than their peers when faced with a novel word to decode. Beyond RTs, Yeatman and colleagues (2021) demonstrated that LDT response accuracy is a promising measure of word recognition ability, across a broad range in development. They introduced an optimized item list for an online LDT which correlated highly (*r* = 0.92) with the Woodcock–Johnson IV Letter-Word Identification scores. However, to our knowledge, no previous study has examined how a classical LDT works as a valid measure of reading ability in the school context, nor applied computerized adaptive testing to improve the efficiency of the LDT.

### Developing browser-based computerized adaptive testing

Computerized adaptive testing is an approach to assessment that uses an algorithm to select the most informative item to present to a test-taker based on their previous responses (Green et al., 1984; Lord, 1971; Weiss, 1982; Weiss & Kingsbury, 1984). Generally, the CAT algorithm estimates a trait (e.g., ability level of the participant) after each response and then selects the next item based on that estimate. The advantage of this approach is that it may provide higher precision with fewer items than conventional tests that use fixed item lists. For example, in the case of ROAR, a first-grade student (or low performer) is unlikely to know the word “homogenization,” and therefore, presenting this difficult item to a low performer is not an efficient use of test time. Similarly, a proficient reader can almost certainly recognize consonant–vowel-consonant words (e.g., dog, cat, sit), and responses to easy items contribute very little to an ability estimate for a high performer. The goal of CAT is to adaptively select items that are most informative for the individual’s ability.

Before transforming an assessment from a linear or random ordering format to CAT, it is important to ensure that the assessment will behave as expected without any threats to validity (Thompson & Weiss, 2011). Simulation experiments are commonly used to ensure the feasibility of CAT and determine the specifications and parameters of the algorithm. Monte Carlo simulation methods (Van der Linden & Glas, 2010; Weiss & Kingsbury, 1984) allow researchers to generate response patterns based on the IRT assumptions and examine the CAT performance based on a variety of test conditions (test length, parameter distributions, item bank size, etc.). However, it is also important to consider data from real participants that might behave differently from the idealized simulation. Post hoc simulations allow researchers to simulate the CAT using real data which might contain intricate response patterns, interactions, or anomalies that would not be considered in a Monte Carlo simulation. Together, these two simulation methods are an important foundation for the development of a CAT assessment.

Although CAT has been widely used in large educational assessment programs (e.g., GRE, GMAT, and Duolingo English Test), designing a CAT solution appropriate for both simulation and real-time web deployment for behavioral science and education researchers remains challenging. First, how can we ensure seamless compatibility between the simulation results and actual deployment? The differences in numerical calculations and random seed numbers between various software programs can potentially impact the results. Second, consider a scenario where we are testing 500 students simultaneously at a school with unstable Wi-Fi and low bandwidth. This implies that we should minimize data communications between the client (test-taker’s computer) and server (where the web application is hosted) to avoid interruptions, delays, and errors that might compromise the measure and waste precious instructional time. Unfortunately, most existing adaptive testing solutions, primarily written in R or Python, need to be run on a server that updates CAT parameters by sending trial-by-trial information between the client and server. The bandwidth demands and complicated client–server infrastructure can impede access to digital testing in low-bandwidth environments and many research studies.

To address these challenges, we developed jsCAT (https://github.com/yeatmanlab/jsCAT), an open-source library written in JavaScript—often referred to as the language of the internet—to support fully client-side computer adaptive testing. Currently, jsCAT supports IRT-based (Rasch, 1PL, 2PL, 3PL, and 4PL) CAT applications and is compatible with popular packages for creating browser-based behavioral experiments such as jsPsych (de Leeuw, 2015). jsCAT currently provides two estimators—the maximum likelihood estimator and expected a posteriori—as well as three methods for the selection of the next item: random, maximum Fisher information, and item difficulty closest to theta (ability). In this paper, we demonstrate improvements in the Rapid Online Assessment of Reading by implementing jsCAT to determine item ordering. After establishing that item difficulty in a LDT generalizes across diverse populations, we examine the efficiency of a computer adaptive LDT through a combination of experiments and simulations.

The rest of this paper is structured as follows: In Study 1, we assess the stability of item difficulty parameters of LDT across different school samples, varying by socioeconomic status, age, and language-based learning difficulties. Study 2 leverages the ROAR item bank to design a computer adaptive, browser-based LDT, and evaluates its efficiency through a real-world experiment in which participants were randomly assigned to either adaptive or random item ordering. In Study 3, we examine the convergent validity of ROAR-CAT by comparing it to proctored, in-person, oral reading assessments commonly used by schools to identify struggling readers in first and second grade. Finally, we conclude with a discussion of the implications of our findings, along with limitations and future research directions.

## Study 1: Analysis of parameter invariance

CAT improves measurement precision with fewer items. However, the effectiveness of CAT depends on the assumption of parameter invariance. Hence, examining its effect is crucial. In IRT, parameter invariance (Rupp & Zumbo, 2004, 2006) means that item parameters are consistent for different populations. While we may never observe identical difficulty parameter estimates in practice, ideally, an item that is relatively difficult or easy for students in one school will be similar for another school; i.e., we would like to see a high correlation between item parameters fit based on the data from different samples. However, there are reasons to be concerned that this may not be the case for a lexical decision task: populations of students differing in age, socioeconomic status, educational experience, and language-based learning differences, including dyslexia, might display systematic differences in LDT responses.

Our previous study used IRT to calibrate item difficulty on a lexical decision task, but this initial study only included a relatively small sample of research participants recruited by two universities (Yeatman et al., 2021). The goal of Study 1 is to examine the stability of item difficulty parameters across different school samples that vary in terms of socioeconomic status, age, and language-based learning difficulties. Based on this analysis, we then remove items that have unstable parameters and evaluate whether parameters are sufficiently stable to implement a computer adaptive version of the task.

### Methods

#### Procedures

**Fig. 1:**
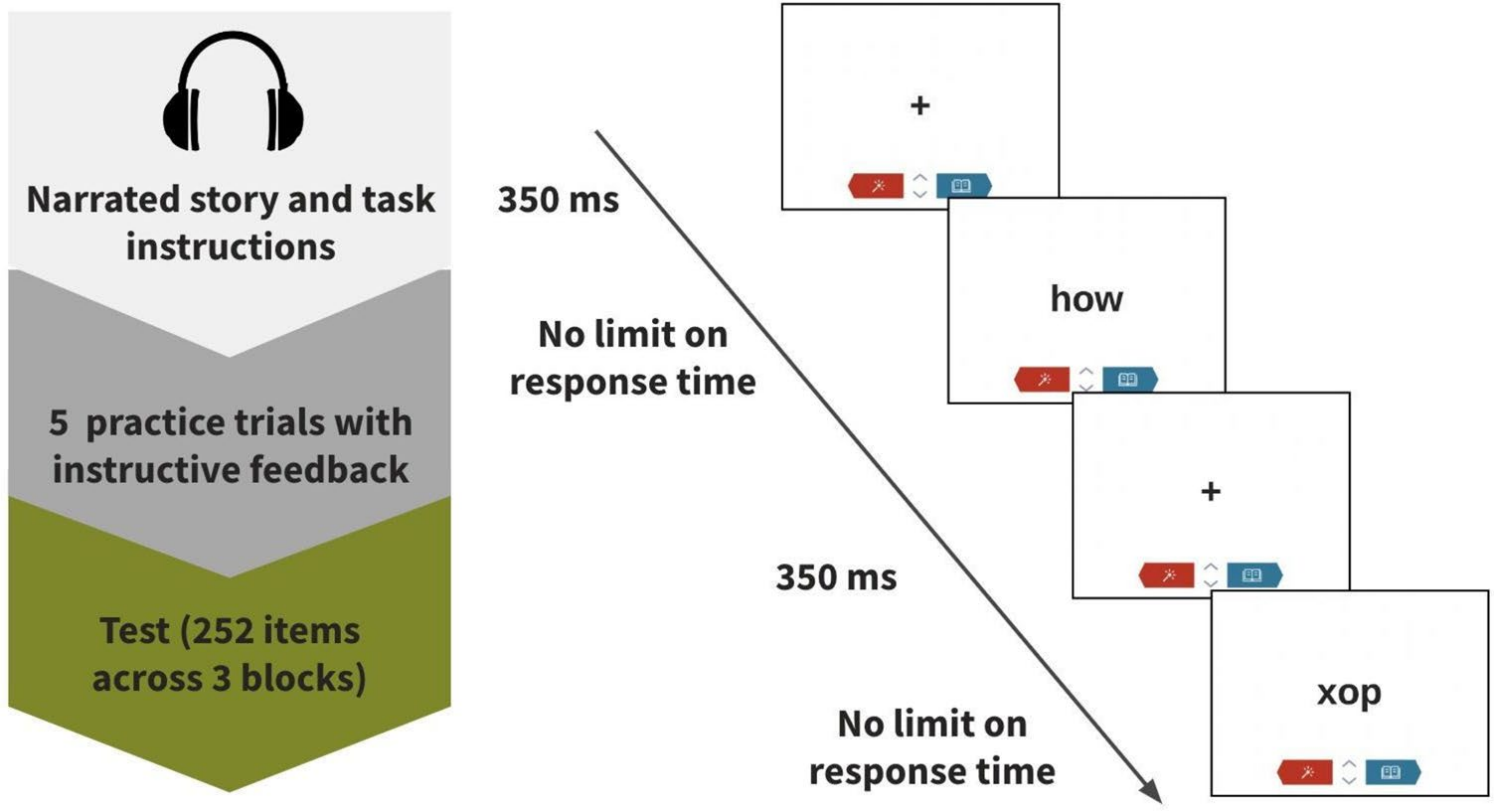
Rapid Online Assessment of Reading Single Word Recognition (ROAR-SWR) trial structure. Task instructions are narrated as part of a story to keep young participants engaged. After completing practice trials with instructions and feedback, participants complete 252 trials of a lexical decision task divided into three blocks

A browser-based LDT was implemented based on the 252 items (which are separated into three test forms consisting of 84 items) that were designed in our previous study and optimized to cover a broad ability range (Fig. 1; see more detail in Yeatman et al., 2021). Each list is presented in a block of trials, the order of the lists is randomized, and the stimuli within each list are presented in a random order (but stimuli were not mixed across lists). Note that we do not use linear fixed-order assessments. Rather, we randomize items to ensure that item parameters do not encode position effects (Kanopka & Domingue, 2022). Each participant was administered the full item bank with 126 real and 126 pseudo words (252 items) that were selected to span a large range of lexical and orthographic properties.

#### Participants

**Fig. 2:**
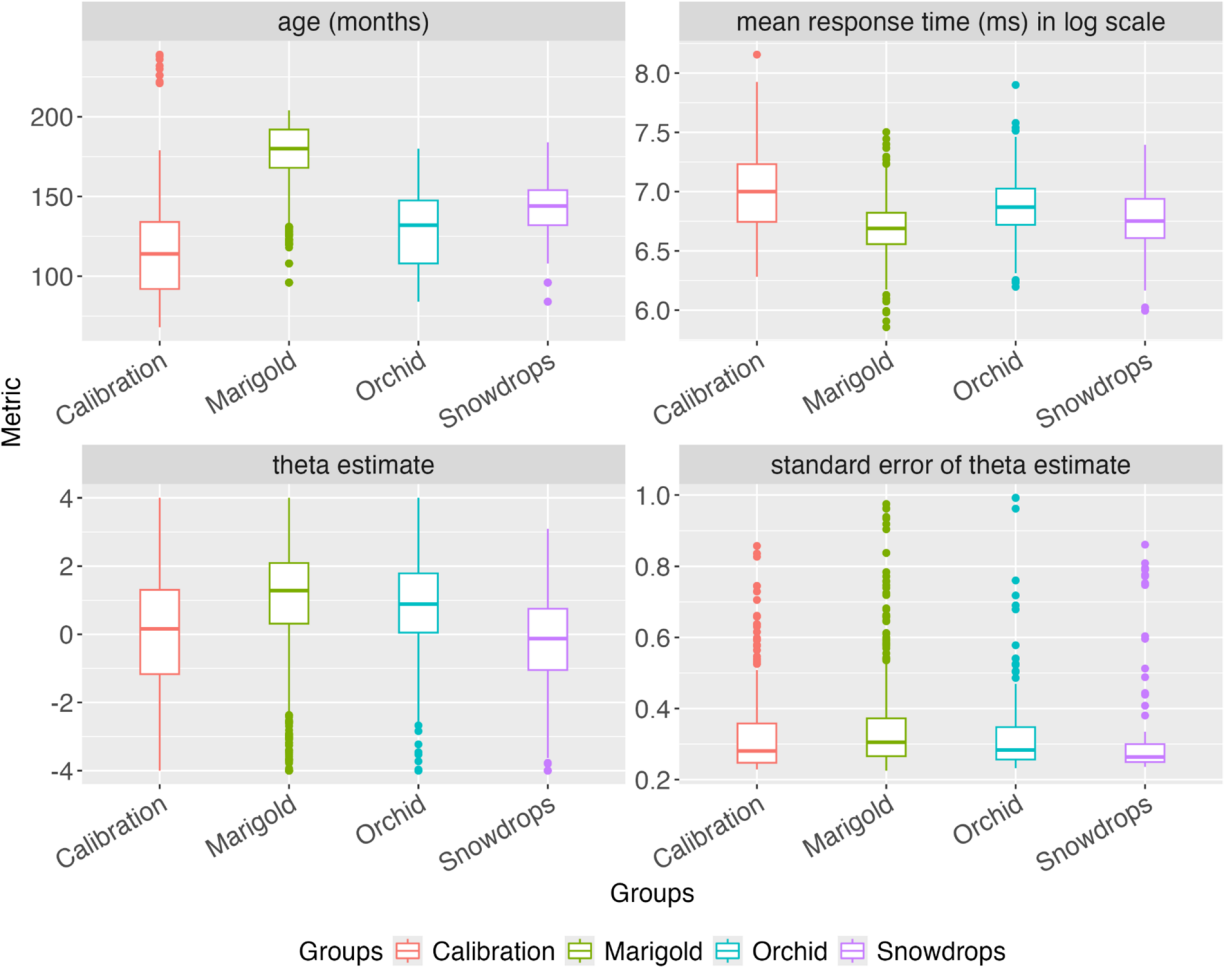
Characteristics of each sample. Box plots show age, response time (RT), ability (theta), and standard error of measurement for each sample. Flower names were assigned to each sample to keep the participating schools anonymous. Ability distributions differ dramatically across schools, highlighting the variability in age and educational experience. For example, the median participant in the Marigold group performed at the 90th percentile of the Snowdrops group

The previous ROAR study (Yeatman et al., 2021) recruited a total of *N* = 306 children and adults through a research participant database (https://dyslexia.stanford.edu/). We refer to this sample that was recruited for university-based research studies as the Calibration group. In the present study, we explored the usefulness of the LDT as a reading ability measure in the school context through the research–practice partnership model (Coburn & Penuel, 2016; Wentworth et al., 2023). The data were collected by partnering with local California school districts as well as private schools that focus on language-based learning disabilities. Each group of school participants was assigned an anonymized flower name, and a description of their sample size and school information can be found in Table 1. The descriptive statistics for each participant group are presented in Fig. 2. The following data analysis only includes participants (*N* = 1960) who completed the LDT for the first time (longitudinal data are excluded). We did not apply additional person-level exclusions, as the focus of this study is on understanding item parameters rather than individual abilities.

**Table 1:** Description of each sample. The Calibration sample was recruited at Stanford University and University of Washington and was described previously (Yeatman et al., 2021). Marigold schools encompassed a relatively low SES public school sample; Orchid schools a relatively high SES public school sample; Snowdrop schools encompassed a private school sample of children with language-based learning disabilities.

Group	N	Description
Calibration	306	Lab-recruited participants
Marigold	1230	Public schools 77% eligible for free and reduced lunch 21% English language learners
Orchid	315	Public schools 11% eligible for free and reduced lunch 6% English language learners
Snowdrop	109	Private schools that focus on language-based learning disabilities Percentage eligible for free and reduced lunch unknown Percentage of English language learners unknown

#### Data analysis

Our previous work (Yeatman et al., 2021) established an item bank appropriately modeled using the 1PL with the lower bound on response probability set at 0.5 (see Eq. 1) based on the Calibration group. In this study, we adopted the same IRT model but we calibrated the item difficulty parameters of the 252 items separately for each group of participants using the mirt package (Chalmers, 2012). The item difficulty parameters (*b*) are estimated on the logit scale, under the default assumption that the mean ability of the participants in the model calibration is 0. Consequently, it is anticipated that items calibrated by a lower-ability group will exhibit relatively higher difficulty parameters than those calibrated by a higher-ability group. Thus, we do not expect the item difficulty values to be the same but, ideally, the ordering of items in terms of difficulty should be consistent across groups (i.e., high correlations between difficulty parameters). To gain an initial understanding of the ability distribution within each participant group, we applied the item parameters derived from the Calibration group to estimate ability scores (theta, *θ*) and standard errors of measurement for each group of students using the maximum likelihood estimator, with the theta range set between − 4 and 4 (see Fig. 2). To calculate the mean response time (RT) for each stimulus on a log scale in milliseconds (ms), we followed established conventions in lexical decision task research, which recognize that extremely fast or slow responses may not accurately reflect participants’ true abilities. Consequently, responses with RTs below 200 ms or above 5000 ms were excluded, resulting in the removal of 3% of the data (14,604 out of 493,920 responses) based on these criteria. This RT-based exclusion criterion was applied only for this specific analysis. For other analyses presented in this paper, we retained all participants’ responses to maintain both the simplicity of the analysis and the representativeness of the student response patterns. To quantify the stability of parameter estimates across samples, we compared the Pearson correlation of the item difficulty parameters and mean RT between the calibration group and three other school groups.

To quantify parameter invariance with the constraints of sample size of different subgroups, we conducted a permutation randomization test (Jorgensen et al., 2018). Post hoc simulations were used to shuffle the groups but vary the exact sample size. Specifically, we randomly assigned each participant to one of the four groups we describe in Table 1 with the probability ratio of 306:1230:315:109, which reflects the sample size per group. Similar to empirical analysis, for each iteration, we estimated the item parameters per group, and then compared the parameter correlations. The final simulation results with 50 iterations were obtained by computing the mean and standard deviation of the correlations; we use these values to calibrate the degree to which observed item difficulty correlations across meaningful groupings diverge as compared to the randomized groupings.

### Results

#### Analysis of parameter invariance

**Fig. 3:**
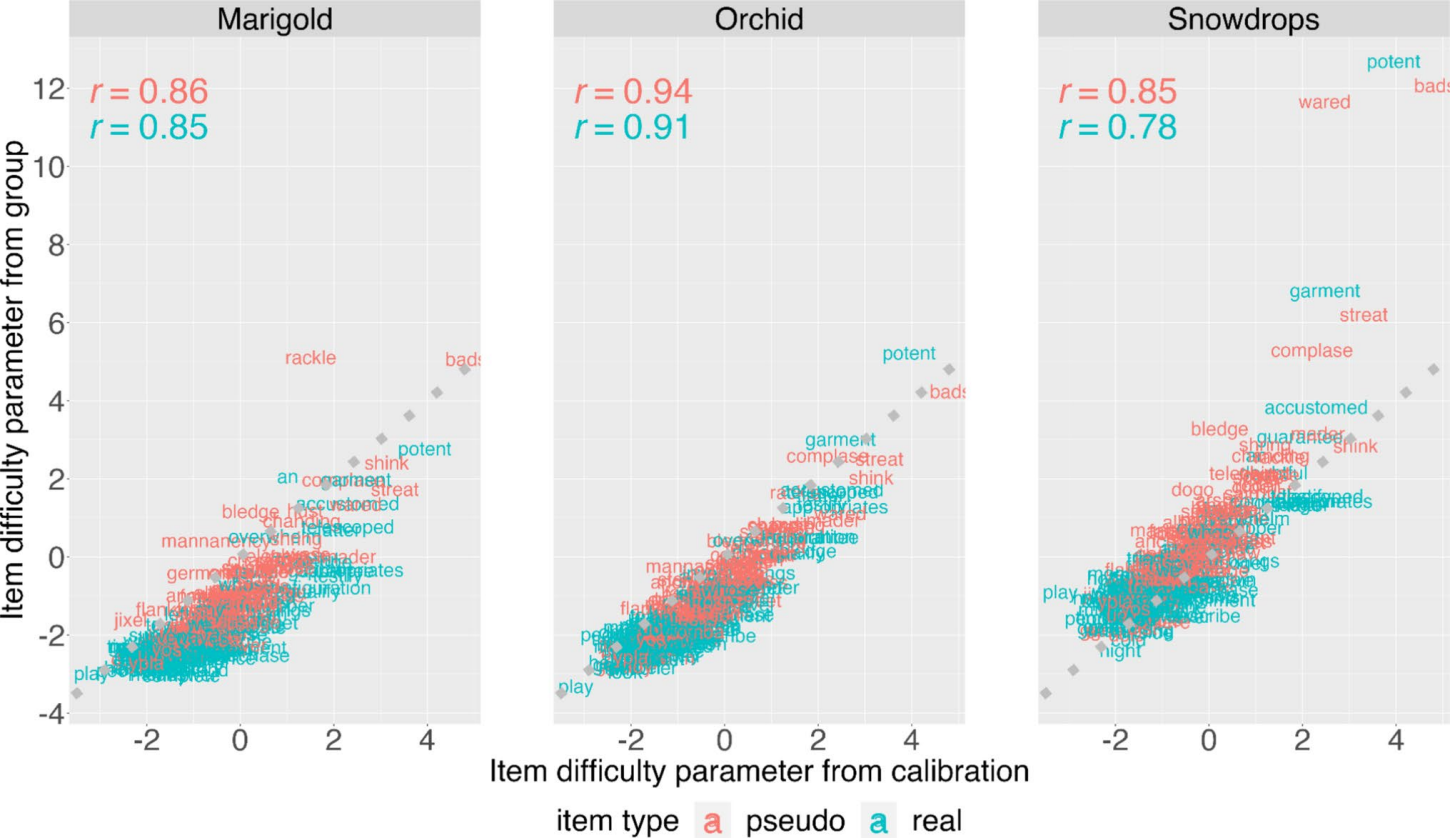
Item difficulty parameters are highly correlated across the four samples. The item difficulty parameters derived from the Calibration group exhibit strong correlations with item difficulty parameters obtained from the three school samples

The item difficulty parameters derived from the Calibration group exhibit strong correlations with item difficulty parameters obtained from the three school samples (Fig. 3)—specifically, for Marigold (all items: *r* = 0.85, real words: *r* = 0.85, pseudo words: *r* = 0.86), Orchid (all items: *r* = 0.93, real words: *r* = 0.91, pseudo words: *r* = 0.94), and Snowdrops (all items: *r* = 0.82, real words: *r* = 0.78, pseudo words: *r* = 0.85). This not only marks a strong start in developing an item bank that can be used across diverse populations but also reveals a compelling finding regarding the stability of the cognitive processes underlying word recognition across diverse populations. For instance, the pseudoword “hust” consistently proved more difficult than “ggnoi,” with a difficulty difference ranging from 3.55 to 3.81, regardless of significant differences in socioeconomic status, age, English language learner status, and language-based learning difficulties among the samples.

In an ideal case with perfect parameter invariance, the correlations would still be less than 1 because the precision of the parameter estimates depends on the sample size (noise ceiling). To determine how far the correlation observed in each sample was from the maximum value that would be expected based on the sample size, we conducted a simulation where all the participants were combined into one group and random samples were drawn based on the size of each group (see Methods). For Orchid (*N* = 315), the mean correlation from the randomization test was *r* = 0.93 (95% CI [0.91, 0.94]), which is identical to the observed correlation, *r* = 0.93. For the Snowdrop group (*N* = 109) and the Calibration group (*N* = 306), the expected mean correlation under perfect parameter invariance was high (*r* = 0.88, 95% CI [0.84, 0.90]). The observed correlation (*r* = 0.82) was only slightly lower than the expected value, reflecting strong alignment in the parameter estimates between students with and without language-based learning differences. For Marigold (*n* = 1230), the randomization test yielded a mean correlation of *r* = 0.96 (95% CI [0.95, 0.97]), exceeding the observed correlation of *r* = 0.85. This discrepancy is examined in the following paragraph. In summary, item difficulty parameters were highly correlated across four samples that differed dramatically in terms of demographic factors, though small differences were observed between parameters for the Marigold and Calibration samples.

**Fig. 4:**
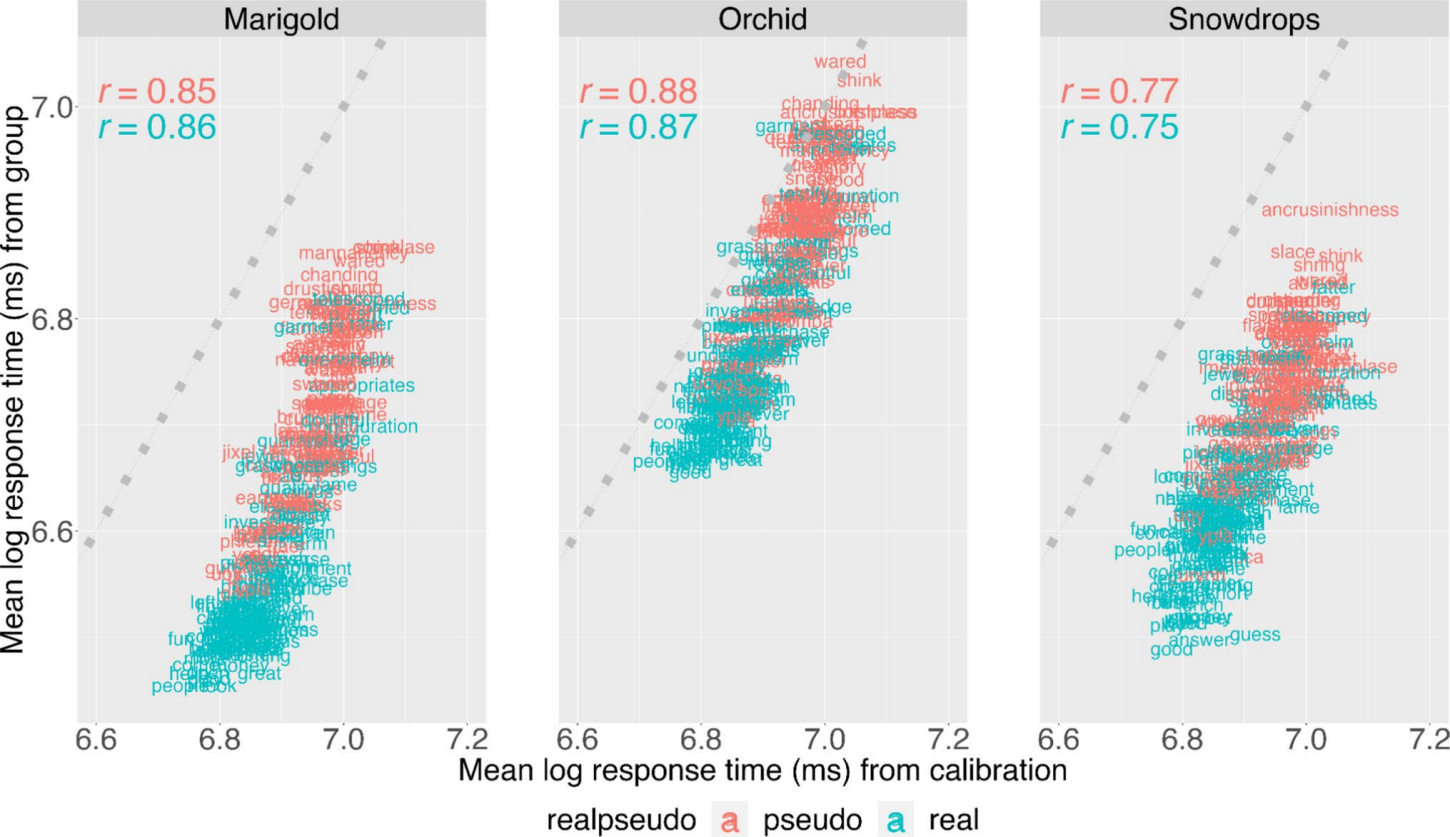
Item response times are highly correlated across the four samples. The mean log RT patterns in milliseconds (ms) from each group are highly correlated with the observed RT patterns from the calibration group

To explain the differences in parameters observed in Marigold, we use RT to understand whether students interacted with the assessment in a similar way beyond correct/incorrect responses. Figure 4 suggests that the mean log RT patterns (ms) from each group are highly correlated with the observed RT patterns from the calibration group. However, the Marigold group, as a whole, responds faster to all items than other groups. This suggests that somewhat different response processes may be used by Marigold students. This difference could be related to the age difference between Marigold students (median age = 15) and those in the calibration sample (median age = 10), Orchid (median age = 11), and Snowdrops (median age = 12). However, even in light of this overall faster RT distribution, item-level RTs were highly correlated, contributing additional evidence for the validity of this measure in diverse samples of school-age children and adolescents.

#### New item bank

While item difficulty estimates were generally quite stable across the four samples, a few items were of concern (Fig. 3). For example, “rackle,” a pseudo word in the item bank, is an outlier that is particularly difficult for the Marigold group (*b* = 4.93) but not others (Calibration: *b* = 1.40, Orchid: *b* = 1.48, Snowdrops: *b* = 2.38). We later found that “rackle” is a Scottish word similar in meaning to “impetuous” in English, so the difference in this item parameter across samples might reflect that high-school students with more English literacy knowledge are more likely to identify it as a less frequent real word. Based on examination of Fig. 3, we removed two items with parameters that varied across populations: “an” and “rackle.” We also removed the two most difficult real words (“potent,” “garment”) and pseudo words (“bads” and “streat”), as they were calibrated as extremely difficult by Snowdrops and therefore do not fit the item bank difficulty distributions.

**Fig. 5:**
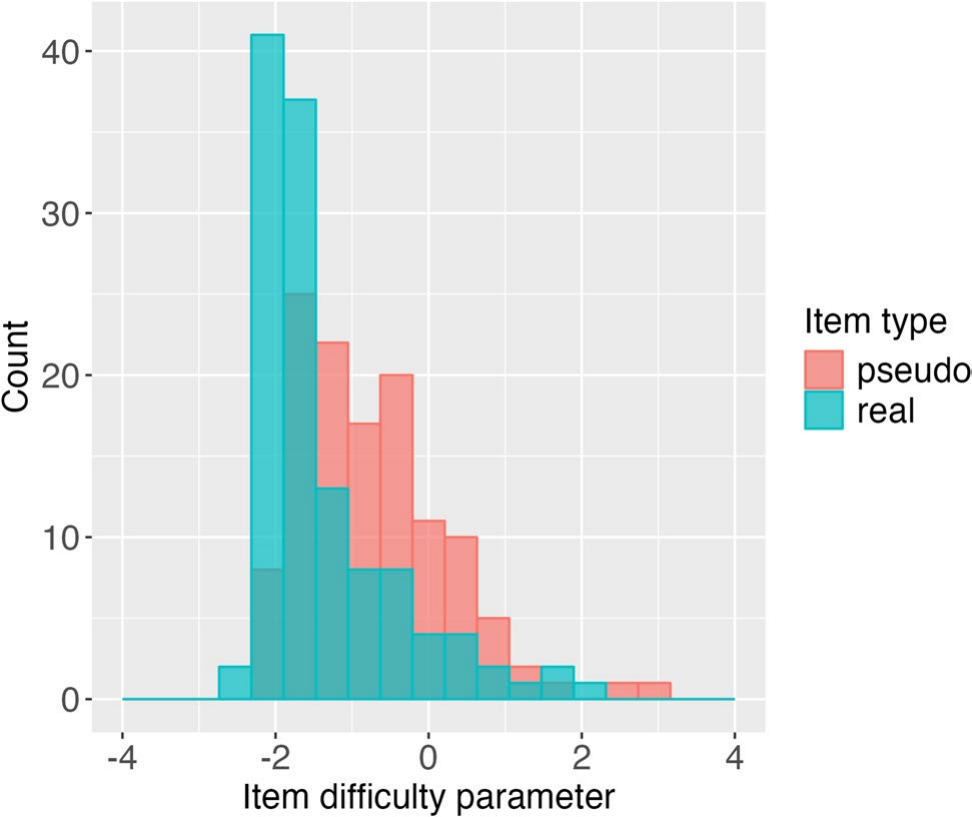
Item difficulty parameters. The new item bank calibrated by the 1PL model with a fixed lower asymptote of 0.5 consisted of 246 items, with an equal distribution of 123 real words and 123 pseudo words

The new item bank consisted of 246 items, with an equal distribution of 123 real words and 123 pseudo words. We incorporated data collected from all the existing lab and school data (*N* = *2*874) and recalibrated the items using the 1PL model with the lower bound on response probability set at 0.5, as shown in Fig. 5. This recalibration was done without accounting for age group effects or other factors. Since parameters were stable across the samples, leveraging the full sample would produce more accurate item parameter estimates for the CAT. It is worth noting that the item bank primarily consists of easier items, indicating its limited ability to distinguish high-ability readers. However, it remains suitable for screening purposes to effectively identify struggling readers.

## Study 2: Validation of ROAR-CAT

Study 1 built the item bank of a simple Rasch scale lexical decision task in ROAR based on diverse school-age cohorts. In Study 2, our objective was to capitalize on the ROAR item bank by designing a computer adaptive, browser-based LDT and to then validate its efficiency through a real-world experiment where participants were randomly assigned to either adaptive or random item ordering. Our primary research questions are as follows:

1. Is CAT item ordering more efficient than random item ordering? If yes, how much efficiency is gained with the present item bank?
2. Are efficiency gains similar or different across the ability range?

In addition to the empirical validation, Monte Carlo simulations were used to compare the empirical efficiency gain with the theoretical efficiency gain. Although CAT is generally more efficient than random ordering, the validation of ROAR-CAT is important given that CAT is only more efficient if certain assumptions hold. These assumptions cannot be determined based on simulations and require real participants. For example, one assumption of IRT is that the order of items does not affect item difficulty. We know this assumption is not completely true. First, item response probability might not be stable when the item is positioned differently in the test (Kanopka & Domingue, 2024). Second, research on lexical decisions (not using IRT) has demonstrated that properties of pseudo words affect decisions on real words (Ratcliff et al., 2004). Imagine an extreme scenario where all the pseudo words were “xxfljmkldamsfd.” In that case, it would be very easy to detect real words. This validation study is essential to connect theory to real human responses. By comparing the empirical data with the simulation results, we can reveal how much of an impact the violation of IRT assumptions has in practice.

We hypothesized that ROAR-CAT would be more efficient than the original random version, and from the results, we hoped to conclude an ideal test length that balances test accuracy and participants’ burden.

### Methods

#### Validation experiment

##### Procedures

We used the jsCAT library to implement a JavaScript CAT algorithm that is compatible with jsPsych and highly comparable to catR (https://github.com/yeatmanlab/jsCAT). Two versions of the LDT task were used for this study: (1) items presented in a random order (ROAR-Random) and (2) item order controlled by jsCAT (ROAR-CAT). Consistent with Study 1, the task was split into three 76-item blocks to give participants a short break in between. We conducted an experiment in which students completed the entire item bank, consisting of 246 items, without a stopping rule. Participants were randomly assigned to either the ROAR-CAT or ROAR-Random condition. Thus, we compared the real-world efficiency of CAT with that of a random ordering of items and also confirmed that CAT did not create any unanticipated bias due to item order effects.

##### Test procedures 

The initial theta is set as 0, and theta is updated after each item using a maximum likelihood estimator with the theta limit (− 4, 4). In the ROAR-CAT group, given a maximum likelihood estimate of theta, the next item is selected based on maximum Fisher information (Van der Linden & Glas, 2000; see Eq. 2) with the counterbalancing criteria between real and pseudo words. The algorithm begins by randomly determining whether the next item will be a real word or a pseudo word, assigning an equal probability of 50% to each choice. Subsequently, it selects the item that provides the maximum information from the remaining items in the current item bank. In the ROAR-Random group, the next item was selected randomly from the item bank with the same counterbalancing criteria between real and pseudo words. We set the test length as 246 items (full item bank), and no extra termination rule was applied. For both versions, the first item is selected from the middle of the item bank, either the real-word item bank or the pseudo word item bank, based on their item difficulty.

##### Evaluation criteria

As a common evaluation practice used in CAT (Wang & Kolen, 2001; Chang & Ying, 1996; Kachergis et al., 2022), we used the standard error of measurement (SEM; see Eq. 3) to evaluate the precision of the measurement based on the IRT model. Additionally, we examined empirical reliability (see Eq. 4) to evaluate the consistency of the measurement: the proportion of observed score variance attributable to true differences in ability (theta) as opposed to measurement error. Furthermore, to evaluate the accuracy of the ability estimates after number of test items ($\widehat{\theta }$) relative to the estimates obtained from the full item bank ($\theta$), we utilized two key metrics: mean squared error (MSE; see Eq. 5) and bias (see Eq. 6). MSE offers a comprehensive measure of prediction accuracy by accounting for both the variance and bias of the estimates, while bias specifically highlights any systematic deviations from the true ability ($\theta$).

Specifically, SEM, reliability, MSE, and bias were calculated after each trial, producing a curve that compares the measurement properties of ROAR-CAT versus ROAR-Random.

2 $$I\left(\theta \right)=\frac{Q\left(\theta , {b}_{i}\right)}{P\left(\theta ,{b}_{i}\right)}{\left(\frac{P\left(\theta ,{b}_{i}\right)-0.5}{1-0.5}\right)}^{2}=4\bullet \frac{1-\left(\theta ,{b}_{i}\right)}{P\left(\theta ,{b}_{i}\right)}\bullet {\left(P\left(\theta ,{b}_{i}\right)-0.5\right)}^{2}$$

3 $$SEM\left(\theta \right)=\sqrt{\frac{1}{I\left(\theta \right)}}$$

4 $${R}_{emp}\left(\theta \right)=\frac{\text{Var}\left(\theta \right)}{\text{Var}\left(\theta \right)+{\text{SEM}\left(\theta \right)}^{2}}=\frac{\frac{1}{N}{\sum }_{i=1}^{N}{\left({\theta }_{i}-\overline{\theta }\right)}^{2}}{\frac{1}{N}{\sum }_{i=1}^{N}{\left({\theta }_{i}-\overline{\theta }\right)}^{2}+{\text{SEM}\left(\theta \right)}^{2}}$$

5 $$MSE\left(\theta \right)=\frac{1}{N}\sum_{i=1}^{N}{\left({\widehat{\theta }}_{i}-{\theta }_{i}\right)}^{2}$$

6 $$bias\left(\theta \right)=\frac{1}{N}\sum_{i=1}^{N}\left({\widehat{\theta }}_{i}-{\theta }_{i}\right)$$

#### Data collection

To validate the ROAR-CAT in a school setting, we ran a study in collaboration with five private schools that focus on learning differences. These schools were in five different states around the country and, therefore, do not represent any specific geographic area. Participants were randomly assigned to ROAR-CAT versus ROAR-Random, allowing us to ascertain how item order affects performance. In total, 485 participants in grades 1–8 participated in this study. Five participants did not complete the assessment, and thus were removed from further analysis. We used the following procedure to identify participants who did not provide data that were an accurate representation of their ability and were removed from further analysis (Yeatman et al., 2021): First, median response times (RTs) in log scale by block were calculated for each participant. Then, participants (*N* = 13) were excluded if the median RT of any of the three blocks was more than three standard deviations below the sample mean. Eight participants (six from ROAR-CAT and two from ROAR-Random) who met this criterion performed the LDT at near chance accuracy level, suggesting that their extremely fast RTs were indicative of random guessing (lack of experiment compliance).

The final dataset includes 229 participants from the ROAR-CAT group and 243 participants from the ROAR-Random group. For each participant, we updated the theta estimate and SEM after each item, and treated the final theta estimate obtained from the full item bank as the ground-truth theta.

#### Monte Carlo simulation

To gain a grounded understanding of the potential efficiency improvements from ROAR-CAT compared to ROAR-Random, given the constraints of the item bank and sample distributions, we conducted Monte Carlo simulations using catR (Magis & Barrada, 2017). Specifically, for each iteration of the simulation, we followed these two steps:

1. Response patterns were generated based on the existing item bank and the final theta distribution from the empirical validation results (*N* = 472).
2. These simulated participants were then randomly assigned to either the ROAR-Random or ROAR-CAT groups, and we calculated the SEM, MSE, and bias correspondingly.

The final simulation results are based on the average of five iterations.

### Results

**Fig. 6:**
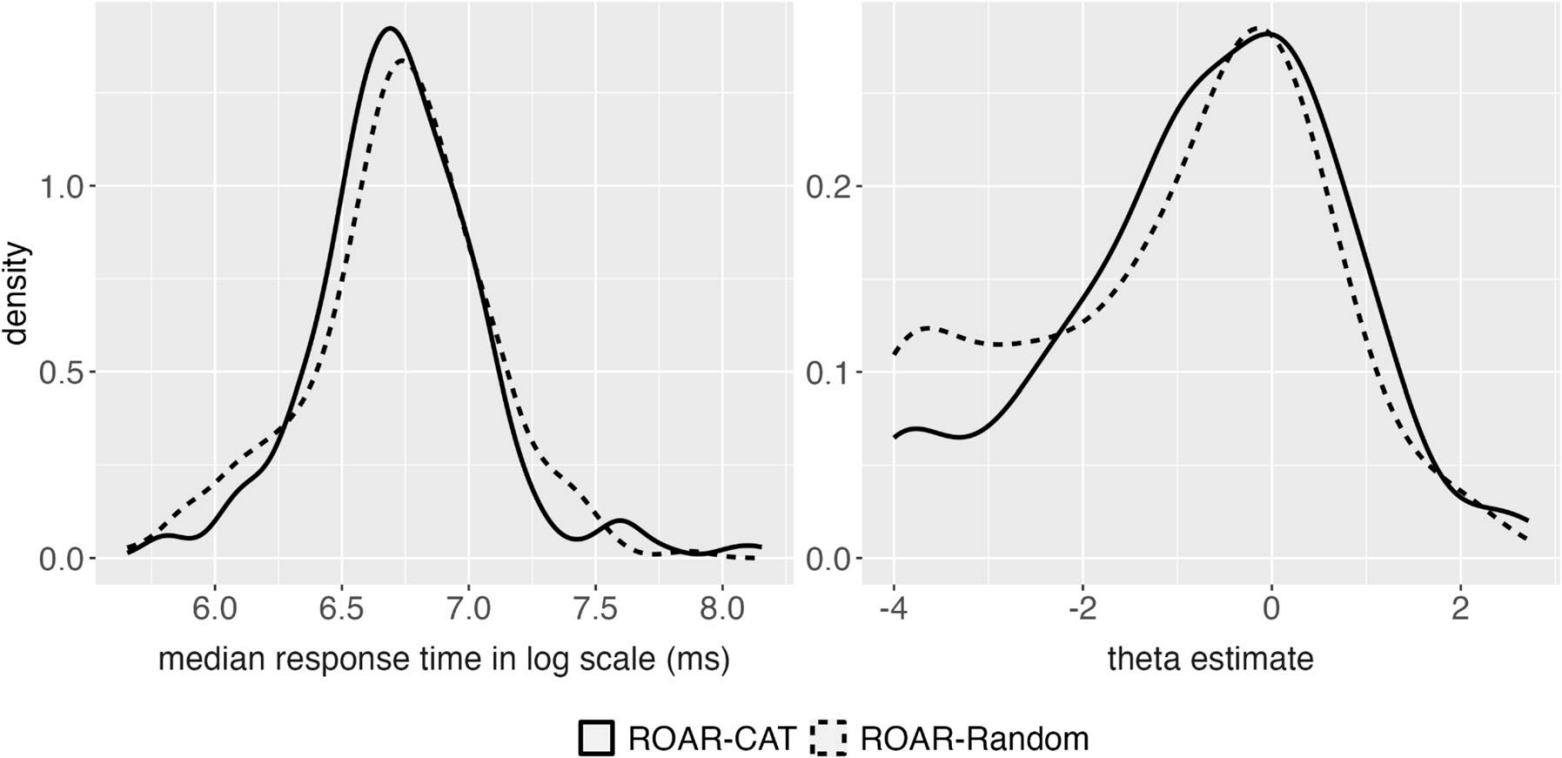
Ability and response time distributions are shown for participants assigned to ROAR-CAT vs. ROAR-Random

Figure 6 displays the distributions of participant abilities and median response times (RTs) obtained from the validation experiment. Despite random assignment, participants assigned to ROAR-CAT (*M* = 137, *SD* = 22) were, on average, 6 months older than those assigned to ROAR-Random (*M* = 131, *SD* = 22; *t*(470) = 3.06, *p* = 0.002). Further analysis demonstrated that participants assigned to ROAR-CAT had slightly higher theta estimates (*N* = 229, *M* =  − 0.73, *SD* = 1.48) than participants assigned to ROAR-Random (*N* = 243, *M* =  − 1.07, *SD* = 1.61), *t*(470) = 2.41, *p* = 0.016). Since participant randomization was implemented in the JavaScript code at the beginning of the experiment, this observed difference reflects true randomness as opposed to a recruitment bias**.** There was no difference in median RT between ROAR-CAT (*M* = 6.73, *SD* = 0.34) and ROAR-Random (*M* = 6.72, *SD* = 0.36). The starting item, selected as the item with median difficulty from the item bank, was fair to both groups. The standard error of measurement for the theta estimate after administering the first item was comparable between the groups: ROAR-CAT (*M* = 13.28, *SD* = 5.52) and ROAR-Random (*M* = 13.34, *SD* = 5.56). It is important to note that the observed abilities of participants in this study are comparatively lower than those seen in Study 1. This distinction can be attributed to the fact that the participants from this validation study are drawn from schools that specialize in educating individuals with language-based learning disabilities.

**Fig. 7:**
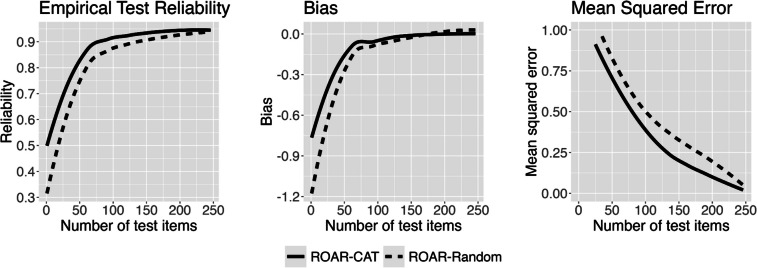
Selecting optimal test length based on validation results. Empirical test reliability, bias, and mean squared error between ROAR-CAT and ROAR-Random

To assess the extent of efficiency improvement brought about by ROAR-CAT in comparison to ROAR-Random, empirical reliability, mean square of error (MSE), and bias were computed at various increments of test items. The empirical results (Fig. 7) suggest that, when considering an equal number of test items, ROAR-CAT (solid lines) is more efficient than ROAR-Random (dotted lines) in providing a precise and accurate theta estimate. As shown in Fig. 7, it becomes evident that ROAR-CAT requires approximately 75 items to achieve reliability of 0.9 while ROAR-Random needs about 125 items, improving efficiency by 40%. The bias converges after approximately 75 test items in both algorithms. With the same number of test items, ROAR-CAT can consistently provide more accurate estimates than ROAR-Random. For example, when test length is set to 100 items, the MSE of final theta estimate decreases from 0.5 with ROAR-Random to 0.375 with ROAR-CAT, reflecting 25% increased accuracy. ROAR-CAT also shows significantly less negative bias than ROAR-Random, especially during the early stage of the test (i.e., first 100 test items).

To explain the effect of the current item bank on the efficiency of ROAR-CAT, we selected three students who were assigned the adaptive version representing a low theta participant (A), high theta participant (B), and medium theta participant (C). Supplementary Fig. 1 shows the progression of ROAR-CAT items, participant responses, and theta estimates. As shown in Supplementary Fig. 1, at an early stage of the test, ROAR-CAT is able to provide items that are very close to the current ability estimate, but after 50 items, the “perfect” item is not available in our item bank. Thus, we expect the efficiency gains to increase with a larger item bank and a more uniform item difficulty distribution.​​​​

**Fig. 8:**
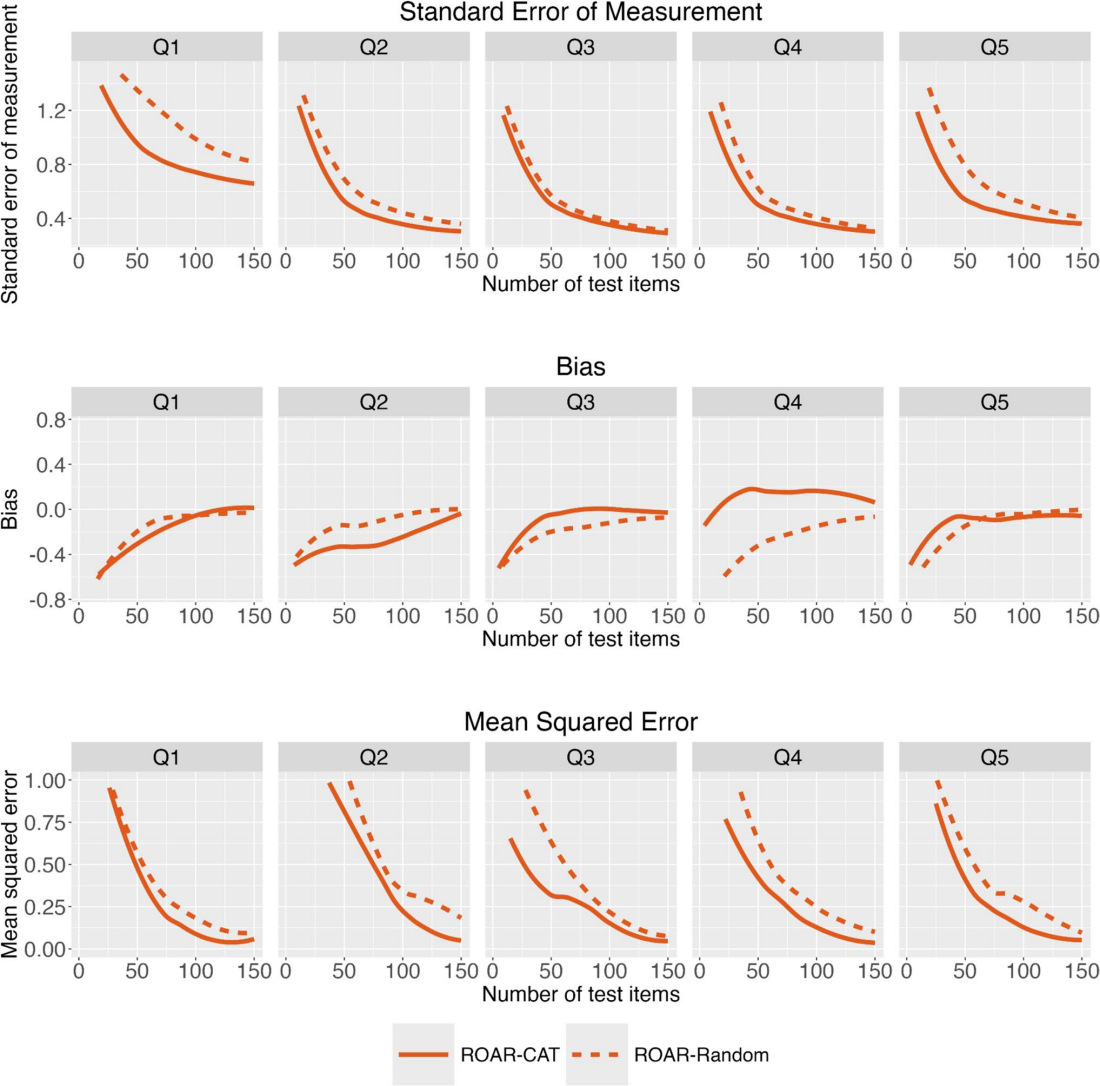
ROAR-CAT validation for participants of different ability levels. Standard error of measurement, bias, and mean squared error between ROAR-CAT and ROAR-Random. Participants were grouped in quintiles by their theta estimates obtained from the full item bank. The cutoff theta estimates for each quintile were as follows: Q1 (− 4, − 2.33), Q2 (− 2.33, − 1.02), Q3 (− 1.02, − 0.26), Q4 (− 0.26, 0.35), Q5 (0.35, 3.27)

To examine the performance of ROAR-CAT in relation to students with varying ability ranges, we categorized participants into quintiles based on their theta estimates from the full item bank (Fig. 8). Q1 represents the lowest theta bin, while Q5 corresponds to the highest. This analysis revealed three interesting observations. First, the standard error of measurement results reveal that ROAR-CAT significantly enhances precision for participants in both the low and high ability quintiles. In the middle quintiles, the differences between ROAR-CAT and ROAR-Random are smaller. This finding is expected based on the current item bank: the item difficulty distribution overlaps most with the abilities of median theta students, so there is a higher probability that a randomly selected item could also be very effectively tailored to their ability (see Fig. 5). Second, in terms of bias metrics, ROAR-CAT exhibits slightly higher bias than ROAR-Random for Q1 and Q2, yet demonstrates lower bias for Q3, Q4, and Q5. Third, the empirical results consistently demonstrate that ROAR-CAT maintains a lower MSE than ROAR-Random across all ability levels. Except for the Q2 bin, ROAR-CAT participants required less than 50 test items to reach an MSE of 0.50 and less than 75 test items to reach an MSE of 0.25.

The Monte Carlo simulation results (Supplementary Fig. 2) serve as a benchmark to confirm that our observations from the empirical results are largely anticipated. First, when examining the standard error of measurement, the simulation results display a precision enhancement comparable to that observed in the empirical data. This consistency suggests that the adaptive algorithm functions reliably even in the highly variable student population in which it was tested. Second, from the simulation results, we observed a bias reduction advantage in ROAR-CAT over ROAR-Random primarily in higher-performing groups (Q4 and Q5). However, empirical data revealed a significant bias reduction advantage not only in these higher-performing groups but also in the middle-performing group (Q3 bin). Third, the simulation results consistently show lower MSE in the ROAR-CAT group than in the ROAR-Random group. However, the simulation performance has lower MSE than the empirical performance. These findings confirm that ROAR-CAT functions as expected, while also suggesting the presence of noise in real human responses. For instance, there may be a slight practice effect in lower-performing groups and increased engagement in adaptive testing when items are precisely matched to students’ abilities. These aspects warrant further investigation in future work with a larger sample size, as well as process (e.g., eye-tracking) and survey data to better measure assessment engagement.

Upon further investigation, we found that the unanticipated inefficiency of the Q2 bin, observed empirically and in contrast to the simulation results, is likely driven by RT choices made by a small group of respondents. We identified a common response pattern among this small group: a significant increase or decrease in RTs as the number of administered items increased, indicating instances of rapid guessing during part of the task. This behavior challenges the validity of their ground-truth theta estimates and is an important point to keep in mind for any online assessment.

Even with the limitation of our item bank and different levels of efficiency gains from students with different abilities, we conclude that ROAR-CAT is more efficient than ROAR-Random in this application, and 75–85 CAT items can achieve a sufficient level of precision and accuracy.

## Study 3: Convergent validity of ROAR-CAT in schools

After validating the improved efficiency of ROAR-CAT, we implemented and deployed an operational ROAR-CAT for large-scale school administration. Study 3 aims to evaluate the convergent validity (Frank et al., 2023) of ROAR-CAT as compared to proctored in-person assessments that schools commonly use for identifying struggling readers in first and second grade. The operational version of ROAR-CAT adheres to the specifications outlined in Study 3, with new items continuously calibrated to the IRT model (for further details, refer to the ROAR Technical Manual: https://roar.stanford.edu/technical). ROAR-CAT is accessible to school partners, families, and researchers by contacting the Stanford ROAR team through roar.stanford.edu or via the standalone platform at https://roar-word.web.app/?recruitment=demo.

### Methods

#### Experimental design

We created a new version of ROAR-CAT that uses 84 adaptive items. For each student, we also collect responses to 16 new items randomly sampled from an uncalibrated item bank (new items: 200 real words and 200 pseudo words). New items were created based on the same procedure as in previous work (Yeatman et al., 2021). The task is split into three blocks (33, 33, and 34 items) to give participants a short break in between. After each five CAT items, the participant will receive a new item. This design enables us to keep calibrating new items without breaking the dynamics of the ROAR-CAT. The theta and measurement error are updated based on the calibrated CAT items only. The CAT design is largely the same as in Study 2. One adjustment we made was that we changed the theta range from (− 4, 4) to (− 6, 6) to enhance measurement precision for participants in the low ability range. This change was prompted by our observation of a substantial number of low-ability participants in Study 2 (see Fig. 6). We anticipate that broadening the theta range will improve the effectiveness of the screener. As demonstrated in Supplementary Fig. 3, expanding the theta range from (− 4, 4) to (− 6, 6) has minimal impact on most theta estimates and the standard error of measurement (SEM), ensuring that the scores remain equitable across different ROAR studies. However, this adjustment provides better differentiation among participants with extremely low ability.

#### Data collection

To assess the value of ROAR-CAT as a screener in a classroom setting compared with other reading assessments that are widely used in schools, we ran a study in collaboration with a collection of California schools to assess the convergent validity of ROAR-CAT against standard-of-practice individually administered reading assessments. Data were collected from first grade and second grade students who completed the ROAR-CAT in spring 2023. Additionally, most of the students were individually administered the Fountas and Pinnell Benchmark Assessment (F&P; Fountas & Pinnell, 2009) by their classroom teacher as part of standard practice, and a small sample of students were also administered the FastBridge Curriculum-Based Measurement for Reading (FAST™ CBMreading; Christ et al, 2018).

To our knowledge, both F&P and FAST™ CBMreading for grades K–2 require educators to complete professional training prior to administration, with assessments conducted individually and in person. In the F&P assessment, students read selected passages aloud individually over a 20–30-min session, after which their reading abilities are categorized into specific levels (AA − Z +). The FAST™ CBMreading involves measuring the number of words read correctly per minute across three passages, with the median score used as the final measure. The total administration time for FAST™ CBMreading is approximately 5 min. It is important to note that although all three assessments—ROAR-CAT, F&P, and FAST™ CBMreading—aim to identify struggling readers, ROAR-CAT uses a completely different format, focusing on a silent lexical decision task rather than oral reading.

#### Data analysis

A total of 351 first grade students and 378 second grade students completed ROAR-CAT in spring 2023. Eleven participants (who had at least two blocks with median log RTs lower than three standard deviations of the mean log RTs) were excluded from the data analysis, as they were identified as rapid guessing. The following analyses are based on the results for 265 first grade students and 241 second grade students who completed ROAR-CAT in spring 2023 and were administered the F&P assessment in March 2023, as well as 42 first grade students and 109 second grade students who completed ROAR-CAT and FAST™ CBMreading in spring 2023. Robust correlations (Wilcox, 2011) were used to compare the relationships between the assessments.

### Results

**Fig. 9:**
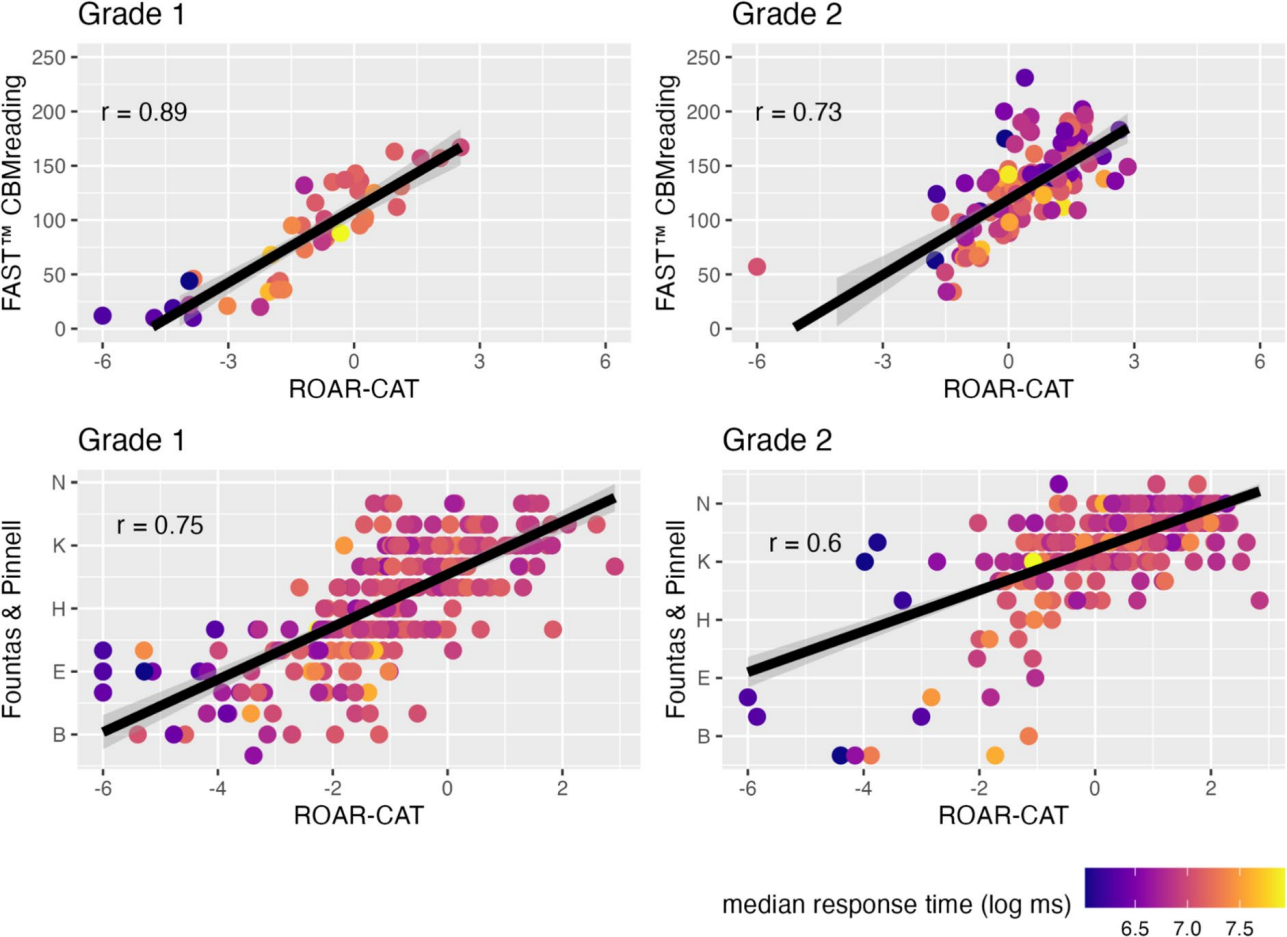
Convergent validity of ROAR-CAT in first and second grade. Comparison among ROAR-CAT, FastBridge Curriculum-Based Measurement for Reading, and Fountas & Pinnell reveals high correlations between the measures. The robust correlation coefficients between ROAR-CAT and FastBridge, and between ROAR-CAT and Fountas & Pinnell are shown on each plot

As shown in Fig. 9, we observed high correlations between ROAR-CAT and FAST™ CBMreading scores: *r* = 0.89 (95% CI [0.82, 0.94]) in first grade and *r* = 0.73 (95% CI [0.62, 0.81]) in second grade. Correlations between ROAR-CAT and F&P were lower than with FAST™ CBMreading but still relatively large: *r* = 0.75 (95% CI [0.69, 0.80]) in first grade and *r* = 0.60 (95% CI [0.51, 0.69]) in second grade. This difference in correlation between ROAR-CAT and FAST™ CBMreading versus ROAR-CAT and F&P is likely due to substantive differences between these measures. FAST™ CBMreading uses test forms that require individual administration and scoring by teachers using electronic devices or paper-and-pencil, whereas F&P is a running record where students read leveled text out loud and a teacher scores them on various scales. The observed differences in correlation between first and second grade likely stem from the differing focus of ROAR-CAT compared to the other two measures, which assess both word recognition skills and oral reading fluency. As oral reading fluency becomes more developed in second grade, it is expected that greater divergences will emerge between ROAR-CAT and the other measures.

**Fig. 10:**
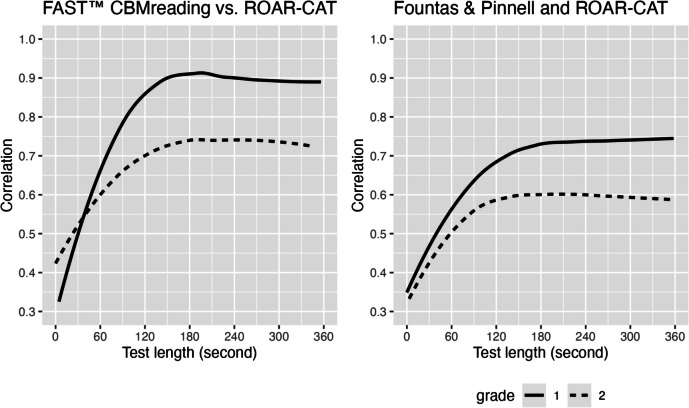
The relationship between test length in seconds and the robust correlations between ROAR-CAT and FAST™ CBMreading, and between ROAR-CAT and Fountas & Pinnell

By plotting the correlations as a function of test length in seconds (see Fig. 10), we observed that the correlations began to stabilize at approximately 180 seconds. This suggests that a 3-min ROAR-CAT is sufficient to predict FAST™ CBMreading and F&P scores.

## Discussion

In this paper, we developed and validated ROAR-CAT for efficient and precise measurement of single-word recognition that is completely automated and can be deployed at scale in research and practice. This is the first study, to our knowledge, to develop a lexical decision task (LDT) in a computerized adaptive format, by leveraging item response theory and the diverse student response data. Our findings from Study 1 suggest that the item parameters for both real and pseudo words, calibrated by a one-parameter logistic model with a fixed lower asymptote at 0.5, remain stable across students varying in age, socioeconomic status, language background, and learning disability diagnosis, including dyslexia. The consistency in LDT item parameters suggests that a CAT version of this task will be both valid and efficient for a diversity of learners. Moreover, we can continually augment the item bank through online item calibration and by monitoring scale drift (Stocking, 1988), further enhancing the efficiency of the CAT.

Study 2 evaluated the increased efficiency and precision achieved by transitioning from randomly ordered items to an adaptive format. This efficient, open-access, browser-based measurement paradigm (lasting 3 to 5 min) will give researchers new opportunities to study visual word recognition (different word patterns, priming effects, speed–accuracy trade-off, etc.) in the context of development and with large-scale representative student samples. It is important to note that our ROAR-CAT validation was based only on students from schools for those with language-based learning differences. We found a significant improvement using adaptive LDT compared with randomly ordered LDT, but it is important to see how well this result generalizes to other populations. To address this question, future work can employ within-subject designs to test how a student responds in different test formats, as well as in a combination of adaptive and random ordering.

Our CAT validation used the most basic algorithm: maximum Fisher information. This is an important first proof-of-concept study, but future work can explore innovations (Chang, 2015) including multidimensional CAT, early stopping rules (Choi et al., 2010), and item exposure control (Barrada et al., 2008) in CAT, cognitive diagnostic CAT (Cheng, 2009), and multistage testing—all of which could offer significant advantages. A clear limitation of the current work is the relatively small size of our calibrated item bank, which hinders the application of more advanced CAT techniques. This shortcoming can be overcome by continually calibrating more items: ROAR-CAT functions as a calibration engine, interleaving validated items for scoring with new items randomly sampled from an uncalibrated item bank. Thus, over time the item bank will grow, allowing for the evaluation of new innovations in CAT algorithms.

In Study 3, we deployed ROAR-CAT in a school setting and demonstrated that theta estimates from ROAR-CAT were highly correlated with proctored in-person oral reading assessments that schools commonly use for identifying struggling readers in first and second grade. These results highlight the potential of ROAR-CAT as an effective screening tool for assessing students' word recognition abilities. Moreover, these results suggest that silent measures like a lexical decision task tap into the same latent construct as oral reading measures. Future research can specifically investigate the utility of ROAR-CAT, potentially in combination with other measures of phonological awareness (Gijbels et al., 2024), sentence reading efficiency (Yeatman et al., 2024), and rapid visual processing (Ramamurthy et al., 2024) as a dyslexia screener. We note that the convergent validity data presented here are based on first grade and second grade only. Our previous work validated ROAR in a broader age range but with a relatively small sample. Thus, it is important for future work to establish convergent validity against more measures and across a larger age range. Longitudinal evidence is also needed to assess the predictive validity of ROAR-CAT as a screener, as well as its efficiency in capturing developmental changes and monitoring progress from interventions and instructional practices.

## Supplementary Information

Below is the link to the electronic supplementary material.

Supplementary file1 (DOCX 815 KB)

## Data Availability

Data available on request for research collaboration purposes.

## Code availability

1. jsCAT software can be found in https://github.com/yeatmanlab/jsCAT

2. The demo of the operational ROAR-CAT (84 adaptive items): https://roar-word.web.app/?recruitment=demo

3. Code to produce the results and figures can be found in https://github.com/yeatmanlab/ROAR-CAT-Public

## Declarations

### Open practices statement

Data available on request for research collaboration purposes, and none of the experiments was preregistered.
