# Supplementary material for: ROAR-CAT: Rapid Online Assessment of Reading ability with Computerized Adaptive Testing
Source: Behav Res Methods. 2025 Jan 14;57(1):56. doi: 10.3758/s13428-024-02578-y (PMC11732908; doi:10.3758/s13428-024-02578-y)
Supplement: Supplementary file 1 — Supplementary file1 (DOCX 815 KB) [file 13428_2024_2578_MOESM1_ESM.docx]

# Supplementary


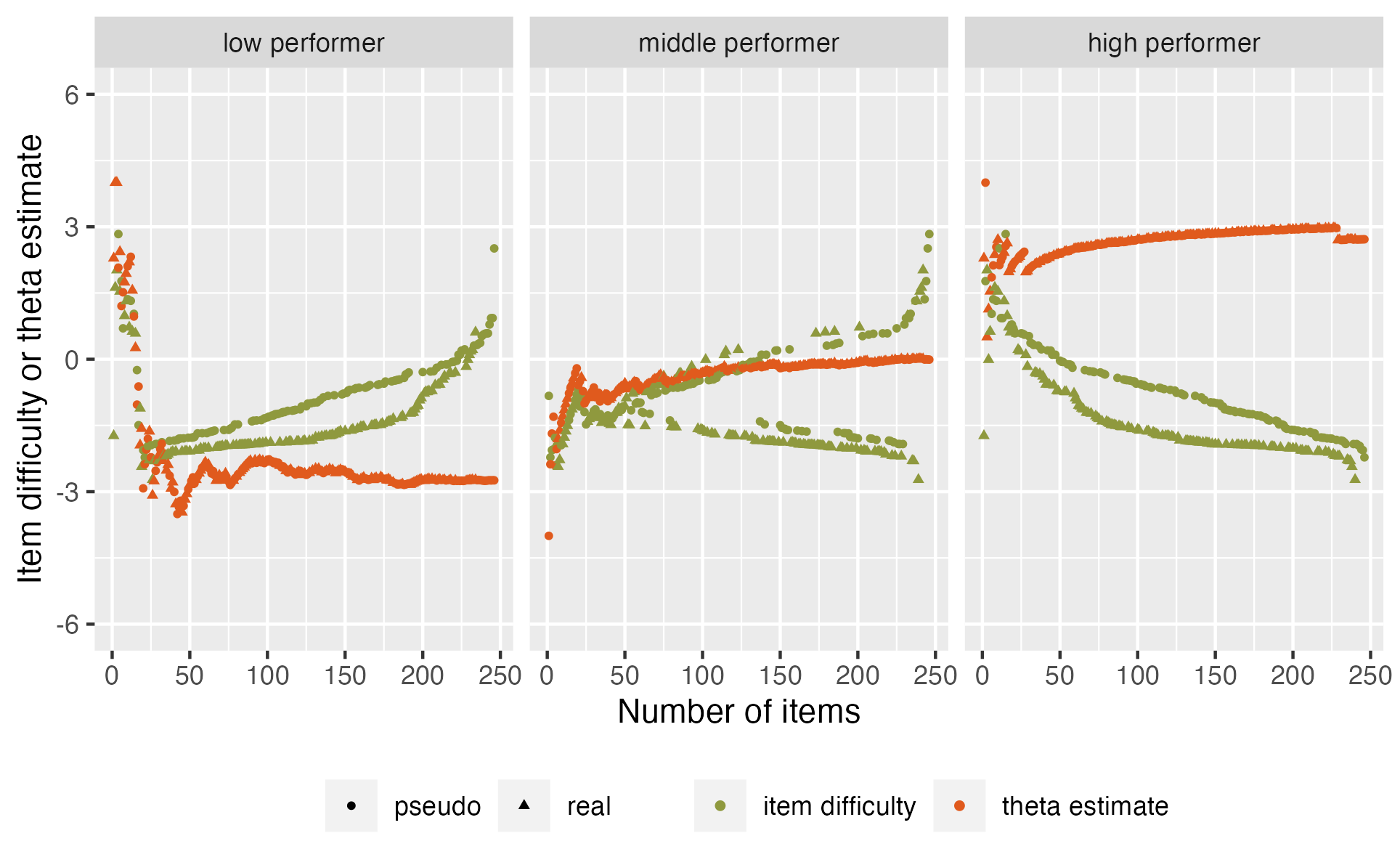


***Supplementary Figure 1: Relationship between theta estimate and best item available as number of items increases.*** *Three students who were assigned the ROAR-CAT are selected to represent a typical trial sequence for low, middle, and high performers. At an early stage of the test, ROAR-CAT is able to provide items that are very close to the current ability estimate, but after around 50 items, the “perfect” item isn’t available in the item bank.*

**
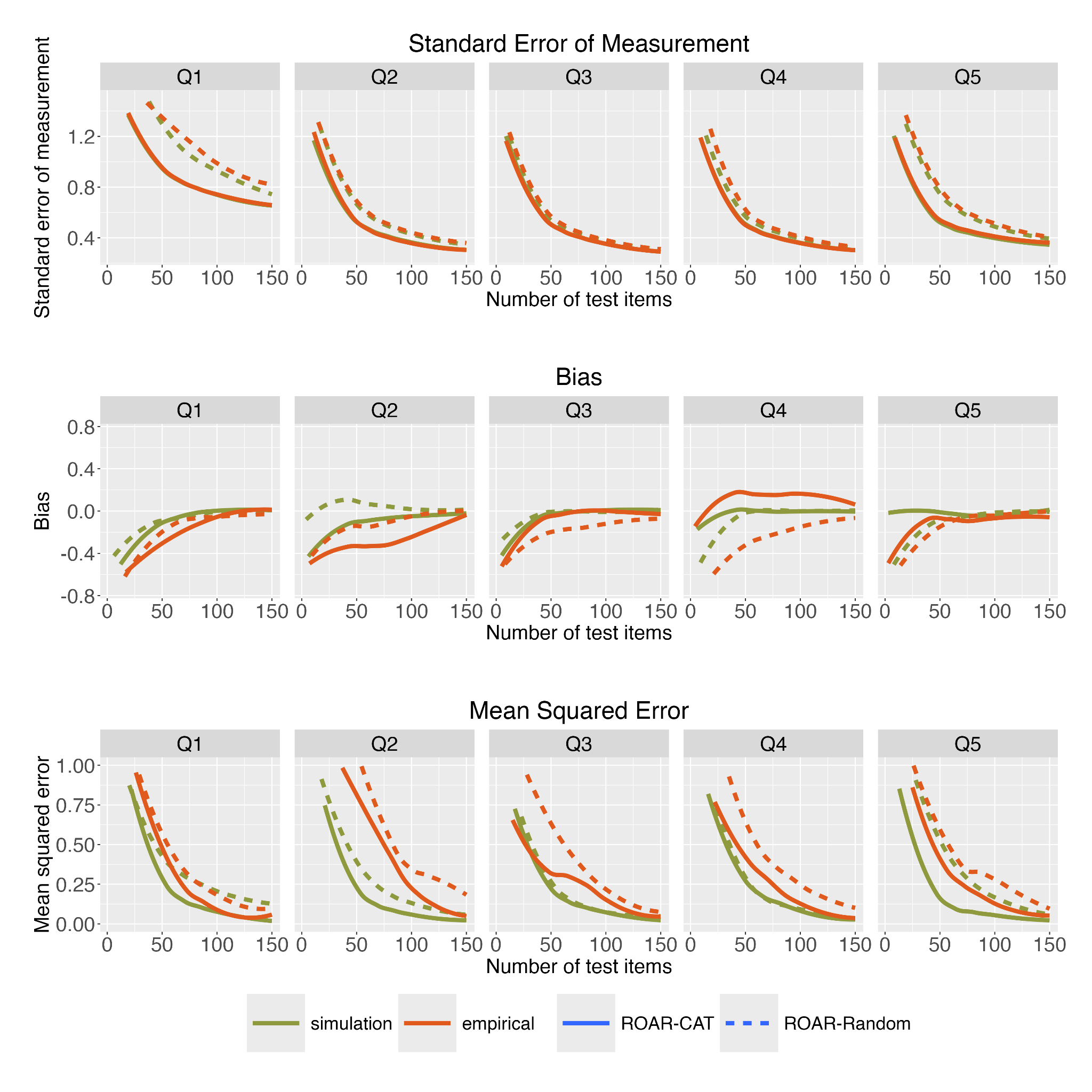
**

***Supplementary Figure 2: Comparison between Monte Carlo simulation results and empirical results of ROAR-CAT validation.***

*
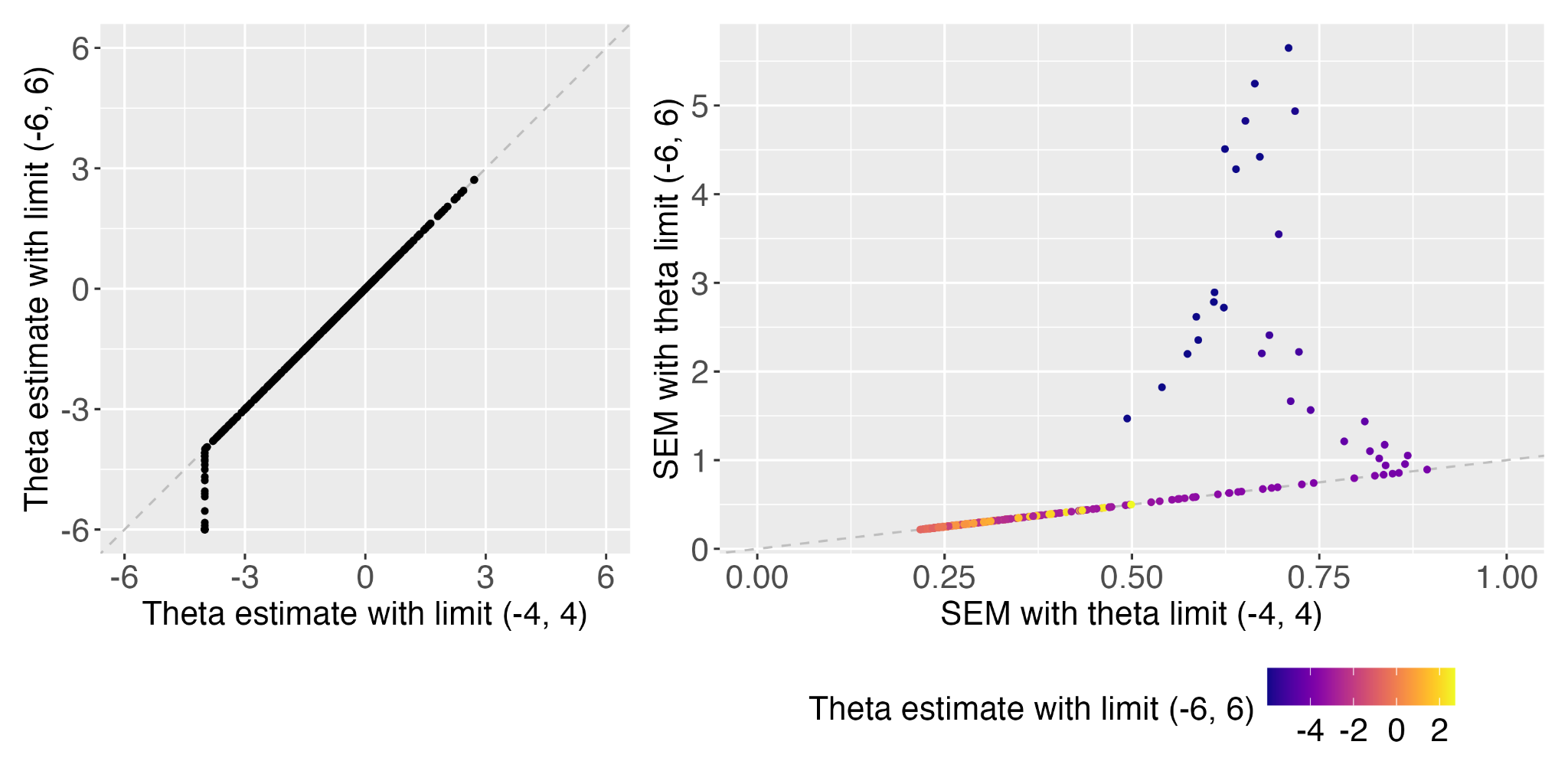
*

***Supplementary Figure 3: Comparison of theta estimate ranges: (-4, 4) vs. (-6, 6) using real data from Study 2.*** *The plot on the left illustrates that expanding the theta range from (-4, 4) to (-6, 6) does not significantly impact most theta estimates. However, it improves the precision of estimates for participants with low ability levels. The plot on the right shows that while the standard error of measurement (SEM) remains largely consistent for most participants, the adjustment in theta range increases the SEM for those with extremely low ability.*
